# Supplementary material for: Assessing operational readiness: Regulatory landscape and compliance in zimbabwe for medical devices and in vitro diagnostic medical devices
Source: PLoS One. 2024 May 16;19(5):e0287495. doi: 10.1371/journal.pone.0287495 (PMC11098427; doi:10.1371/journal.pone.0287495)
Supplement: Attachment — Submitted filename: Review.pdf [file pone.0287495.s002.pdf]

Medical Devises Regulation in Zimbabwe: An Evaluation of operational readiness

Manuscript ID: PONE-D-23-17232

Summary of the research and overall impression:

The study reports an analysis of the readiness of medical device regulation in Zimbabwe according to the Global Benchmarking Tool+ provided by World Health Organization. The manuscript highlights how regulation of medical devices has seriously lagged not only in Zimbabwe but in low-income countries compared to EU regulation or FDA in US. Results report that, according to the proposed framework, maturity level of medical devices regulation in Zimbabwe is 1, underlining the lack of guidelines by regulatory agencies in all the phases of the management cycle of medical devices including post-market surveillance and control. In the opinion of this reviewer the article topic is really relevant. Technical aspects of the study design and data analysis are well detailed and documented. However, some parts need to be rearranged in a more accessible way.

The authors should address the following issues:

Major Revisions

1. Please correct the misspelling in the title about the "devises" word.
2. Please review the English grammar and vocabulary in the entire manuscript.
3. Some concepts are repeated a lot of times, with same words and expressions. Please consider eliminating repetitions especially in the results discussion section.
4. An extensive number of acronyms are used in the text, although they are necessary a large number of acronyms make the manuscript difficult to read. It is advisable to avoid them where possible. Some acronyms are not necessary because they are not frequently recalled in the text.

Minor Revisions:

5. Image captions are very poor in content. Please include more information about reported graphs in figure 1 and figure 2.
6. Line 10-11: Please rephrase "There are disparities in regulating medical devices; in the African region, it is below the global average".
7. Line 67: Developing countries is a term disused. Please substitute with low-income countries or any term you consider appropriate to this context.
8. Line 93-94: "About 40% of countries in the WHO Afro region had no regulations for medical devices, 32% had some regulations, and 28% had no regulations". Please rephrase, it is not clear how percentages are distributed.
9. Line 123: Include in the manuscript NRAs acronym definition. It is present only in the abstract.
10. Line 301-302: "No requirements require medical devices.." Please rephrase.
11. Line 350: "RS" please avoid the use of acronyms also in the titles.
12. Line 378: Please instead of cite the single author (Dube-Mwedzi) cite the published journal article.

13. Line 401: “MA” please avoid the use of acronyms also in the titles.
14. Line 406: Please consider that GHTF is the acronym referring to global harmonization task force, not the global harmonization working party.
15. Line 418: “VL” please avoid the use of acronyms also in the titles.
16. Line 439: “MC” please avoid the use of acronyms also in the titles.
17. Line 461-462: “Still they were not doing so” please rephrase.
18. Line 462: US acronym is already used. Please redefine at the beginning of the manuscript. Same suggestion can be addressed for European Union.
19. Line 468: “LI” please avoid the use of acronyms also in the titles.
20. Line 478: “LT” please avoid the use of acronyms also in the titles.
21. Line 480: Please rephrase clarifying what it is included what it is not.
22. Line 492: Please also insert the year in the ISO standards.
23. Line 495: Please also insert the year in the ISO standard.
24. Line 503: “RI” please avoid the use of acronyms also in the titles.
25. Line 514: “CT” please avoid the use of acronyms also in the titles.
26. When regulation are cited, it is better to refer directly to the regulation instead of a document reporting the regulation.
27. Citations 14 and 15 are the same paper.
28. Please check that the citation style is uniform.
