# Supplementary material for: Assessing operational readiness: Regulatory landscape and compliance in zimbabwe for medical devices and in vitro diagnostic medical devices
Source: PLoS One. 2024 May 16;19(5):e0287495. doi: 10.1371/journal.pone.0287495 (PMC11098427; doi:10.1371/journal.pone.0287495)
Supplement: Attachment — Submitted filename: Review2.pdf [file pone.0287495.s004.pdf]

Medical Devises Regulation in Zimbabwe: An Evaluation of operational readiness

Manuscript ID: PONE-D-23-17232

The reviewer thanks the authors for addressing the revisions suggested. However, the authors should address the following issues:

Major revisions:

1. Line 275: "Two participants were interviewed and assisted with information to complete the questionnaires administered by the researcher." Were only two participants interviewed? Which are the inclusion criteria on which they were chosen?
2. Introduction section is still long. The reviewer's suggestion is to maintain only the information necessary to have an overview of the problem addressed by the manuscript.
3. Grammar revision has been not effective. Here some examples:
   "Medical devices are essential for the public to meet their highest health standards" instead of "Medical devices are essential for the public to reach the highest health standards."

   "Medical Device Regulations are a set of laws and regulations governing the clinical trials, manufacturing, and distribution of medical devices to ensure that they are safe and perform as intended by their manufacturers" Instead of "Medical Device Regulations are a set of laws and regulations governing clinical trials, manufacturing, and distribution of medical devices ensure they are safe and perform as intended by their manufacturers".

   "Participation in the study was voluntary" instead of "Participation was voluntary"

   "The participants were able to stop the interview at any time without any explanation" instead of "Participants were able to stop the interview at any time without explanation"

   These examples are not exhaustive.

Minor revisions:

4. Image 1 is low quality. In addition, in the opinion of the reviewer the image does not add any value to the manuscript.
5. Line 117-118: "In 2016, the World Health Organization (WHO) conducted a 117 review to evaluate the availability of medical device regulations in Africa." Are you sure only Africa was the target area of the study? Probably you are referring to WHO regulatory status desk survey (July 2016 update) and this study was targeted to map all the world status. Please even if this is not the study you have analyzed, cite in the text which document you are referring to.
6. Line 146: " countries of the Surgeons of Central, Eastern and Southern Africa (COSECA)", the acronym was already defined.
7. Line 228: "The researcher interviewed the staff responsible for the regulations and conducted a desk reviewed the documents." Please check the grammar or rephrase.
8. Line 548: Please insert the year of the standard.
9. The reviewer suggests to cut all the parts of the manuscript that really do not refer to the main scope and to insert them as supplementary material.

10. Answer to comment 26 of the first review. Reference n.2 (GHTF. Global Harmonization Task Force Study Group 1: Definition of the Terms 'Medical Device' and 'In Vitro Diagnostic (IVD) Medical Device.' Force, Study Gr 1Glob Harmon Task [Internet]. 2012;(Ivd):6. Available from:http://www.imdrf.org/docs/ghtf/final/sg1/technical-docs/ghtf-sg1-n071-2012 definition-of-terms-120516.pdf#search=%22ghtf definition ?Medical Device?
    is not necessary as you can refer directly to MDR 745/2017 and IVDR 746/2017 for the definitions of medical device and in-vitro diagnostic medical device.

Line numbers refer to the version without track changes.
