# Supplementary material for: Assessing operational readiness: Regulatory landscape and compliance in zimbabwe for medical devices and in vitro diagnostic medical devices
Source: PLoS One. 2024 May 16;19(5):e0287495. doi: 10.1371/journal.pone.0287495 (PMC11098427; doi:10.1371/journal.pone.0287495)
Supplement: Attachment — Submitted filename: Response to Reviewers comments .docx [file pone.0287495.s003.docx]

**Charles CHIKU**

**419 Rydale Ridge Park,**

**Harare, Zimbabwe**

**Email address: [charleschiku@gmail.com](mailto:charleschiku@gmail.com)**

**+41783450910**

**29 October 2023**

**Zenewton André da Silva Gama, Ph.D.**

**Academic Editor**

**PLOS ONE**

**Response to Reviewers’ comments for the study PONE-D-23-17232**

**Medical Devises Regulation in Zimbabwe: An Evaluation of operational readiness.**

Thank you very much for your time and effort in reviewing the manuscript with the title on the subject line. I am grateful for the invaluable feedback you and the other reviewers provided to improve my work. I responded to each question raised during the review process. Where I needed to explain the rationale for the approach I used, I have done so as well. Each question had a corresponding response. The questions from the reviewers are indented and in bold to distinguish them from the responses provided.

**General Comments in Email**

***1. Please ensure that your manuscript meets PLOS ONE's style requirements, including those for file naming. The PLOS ONE style templates can be found at***

***https://journals.plos.org/plosone/s/file?id=wjVg/PLOSOne_formatting_sample_main_body.pdf and***

[***https://journals.plos.org/plosone/s/file?id=ba62/PLOSOne_formatting_sample_title_authors_affiliations.pdf***](https://journals.plos.org/plosone/s/file?id=ba62/PLOSOne_formatting_sample_title_authors_affiliations.pdf)

Response: The manuscript was edited to meet PLOS ONE’S style requirements, including file naming requirements.

***2***. ***Note from Emily Chenette, Editor in Chief of PLOS ONE, and Iain Hrynaszkiewicz, Director of Open Research Solutions at PLOS: Did you know that depositing data in a repository is associated with up to a 25% citation advantage (https://doi.org/10.1371/journal.pone.0230416)? If you’ve not already done so, consider depositing your raw data in a repository to ensure your work is read, appreciated and cited by the largest possible audience. You’ll also earn an Accessible Data icon on your published paper if you deposit your data in any participating repository (***[***https://plos.org/open-science/open-data/#accessible-data***](https://plos.org/open-science/open-data/#accessible-data)***).***

Response: Data was deposited in Open Science Framework (OSF) Registries under the Project name Medical devices regulation in Zimbabwe: an evaluation of operational readiness, doi: <https://doi.org/10.17605/OSF.IO/C5JXG>.

***3. Please include captions for your Supporting Information files at the end of your manuscript and update any in-text citations to match accordingly. Please see our Supporting Information guidelines for more information:*** [***http://journals.plos.org/plosone/s/supporting-information***](http://journals.plos.org/plosone/s/supporting-information)***.***

Response: There are no supporting information files attached to the manuscript. The files added in the initial submission as Supporting files 1 and Supporting file 2 were raw data. These files were deposited in the OSF Registries at <https://doi.org/10.17605/OSF.IO/C5JXG>.

**Major Revisions**

**Reviewer 1**

***Reviewers’ comment***

1. ***Please correct the misspelling in the title about the “devises” word.***

Response: The spelling is corrected in the title and throughout the manuscript. Please see the title in both the clean and tracked manuscript versions.

***Reviewers’ comment***

2. ***Please review the English grammar and vocabulary in the entire manuscript.***

Response: The entire manuscript was reviewed and corrected to address all the identified English grammar and vocabulary errors identified by the reviewers. The tracked changes version of the manuscript shows the corrections that addressed the grammatical and vocabulary errors.

***Reviewers’ comment***

3. ***Some concepts are repeated a lot of times, with the same words and expressions. Please consider eliminating repetitions, especially in the results and discussion section.***

Response: Repeated concepts, such as registration and market authorization, dealing with substandard and falsified products, were revised, and repetitions were eliminated.

***Reviewers’ comment***

4. ***An extensive number of acronyms are used in the text; although they are necessary, a large number of acronyms make the manuscript difficult to read. It is advisable to avoid them where possible. Some acronyms are not necessary because they are not frequently recalled in the text.***

Response: Acronyms were reduced and referred to regulatory functions in full instead of using acronyms. Please refer to lines 454-572.

**Minor Revisions:**

***Reviewers’ comment***

5. ***Image captions are very poor in content. Please include more information about reported graphs in figure 1 and figure 2.***

Response: Figures 1 and 2 were combined into a single figure, named Fig 1, in the revised submission. Revisions include the legend and caption to give the readers a better understanding of the results. The legend was added in the manuscript just below the title of the figure to meet the requirements of PLOS One journal as stated in the submission guidelines.

***Reviewers’ comment:***

6. ***Line 10-11: Please rephrase“There are disparities in regulating medical devices; in the African region, it is below the global average”.***

Response: The statement was corrected and read as, “There are disparities in the regulation of medical devices in the African region that are below the global average.” (Line 33)*.*

***Reviewers’ comment:***

7. ***Line 67: Developing countries is a term disused. Please substitute with low--income countries or any term you consider appropriate to this context.***

Response: The word developing countries was replaced with RLS (Resource Limited Settings). Lines 90-95, “ It has also been concluded that poor regulatory systems in RLS make it difficult for manufacturers to introduce their products into these markets, resulting in limited access to them.”

**Reviewers’ comment**

8. **Line 93-94: “About 40% of countries in the WHO Afro region had no regulations for medical devices, 32% had some regulations, and 28% had no regulations”. Please rephrase, it is not clear how percentages are distributed.**

Response: This section was reviewed to report the source of information in the context of the desk review conducted by the World Health Organization. Criteria on the classification of regulatory systems in the surveyed member states were also included, as shown below: “ The WHO conducted a desk review in 2016 to assess the availability of medical device legislation in Africa. Countries were classified based on premarket, market, and post-market regulatory elements as follows:

•	Have all the three types of elements,

•	Have premarket and placing on the market elements

•	Have premarket and post-market elements,

•	Have placing on the market and post-market elements

•	Have only premarket elements

•	Have only placing on the market elements

•	Do not have any element

•	Data not available (member states did not respond to the survey).

•	Not applicable

In the Afro region, 40% of countries have no medical device regulations, 32% have some regulations, and 28% lack data. Globally, 58% of the WHO members regulate these devices. Zimbabwe was reported as regulating medical device market placement, but data is vague (12) .” (lines 115-130).

***Reviewers’ comment***

9. ***Line 123: Include in the manuscript NRAs acronym definition. It is present only in the abstract.***

Response: The acronym NRA was mentioned in full at the first mention in the body, as shown in lines 202-204, “Manufacturers may be hesitant to introduce their products in countries with weak regulations if the National Regulatory Authority (NRA) has additional requirements compared to mature regulatory authorities, where these products may have been authorized.”

***R*eviewers’ comment**

10. **Line 301-302:“No requirements require medical devices..” Please rephrase.**

Response: The statement was corrected: “Medical devices lack legal compliance for registration and authorization. No conformity assessments were proportional to the risk class of medical devices. " (Lines 366-367).

***Reviewers comment:***

11. ***Line 350: “RS” please avoid the use of acronyms also in the titles.***

Response: Acronyms were removed from all the titles in the results and discussion section of the manuscript as follows: Regulatory System (line 395), Registration and Market Authorisation (line 454), Vigilance (line 477), Market Surveillance and Control (line 507), Licensing Establishment (532), Laboratory Testing (line 543), Regulatory Inspection (line 561), and Clinical Trials Oversight (572).

***Reviewers’ comment***

12. ***Line 378: Please, instead of cite the single author (Dube-Mwedzi), cite the published journal article.***

Response: The journal article was cited as “This agrees with a study conducted by Dube-Mwedzi et al., which found that the regulatory frameworks for medicines were strengthened, while medical devices were not a priority in the rapid assessment of the National Regulatory Systems for medical products in the Southern African Development Community, which clearly shows that Zimbabwe's medical device regulatory system has not been responsive, outcome-oriented, predictable, risk proportionate to public health risk, and independent; these are critical characteristics of a robust regulatory system. This finding is in agreement with that of Dube-Mwedzi et al. (12). Poor quality medical devices have a significant impact on health care outcomes. It is widely accepted that the value of medication relies mainly on the accuracy of diagnosis. Thus, the use of poor-quality medical devices and diagnostics undermines the effectiveness of effective medicines. Investing more effort in strengthening frameworks for medical devices is recommended as a priority for SADC countries.

The methodology used in the rapid assessment mentioned above differed from this study in that Dube-Mwedzi et al.'s focused on the SADC member states, and the data lacked granularity to identify where Zimbabwe stood in the study. In our study, we conducted an in-depth examination of the Zimbabwean context using a global benchmarking tool to assess the quality of implementation of the medical device regulatory system.”(lines 418-435).

***Reviewers’ Comment:***

13. ***Line 401: “MA” please avoid the use of acronyms also in the titles.***

Response: The acronym was replaced with Registration and Market Authorisation (line 454).

***Reviewers’ comment:***

14. ***Line 406: Please consider that GHTF is the acronym referring to global harmonization task force, not the global harmonization working party.***

Response: My apologies for the typographical error. The statement has been corrected as “The International Medical Devices Regulatory Framework (IMDRF) and its predecessor, the Global Harmonization Task Force (GHTF), recommend that medical devices be assigned to one of four classes based on a set of rules.*.” (lines 467-449).*

***Reviewers comment:***

15. ***Line 418: “VL” please avoid the use of acronyms also in the titles.***
16. ***Line 439: “MC” please avoid the use of acronyms also in the titles.***

Response: Please refer to the response to request 11.

***Reviewers’ comment***

17. ***Line 461-462: “Still they were not doing so” please rephrase.***

Response: The statement was rephrased: "One study found that most countries in the region have legal authority to combat substandard and falsified medical products, but implementation is lacking, and challenges in managing medical products have been documented in the SADC region. However, more information is required regarding medical devices, including IVDs. A study conducted to map existing frameworks, mechanisms and approaches to prevention, detection and response to substandard and falsified medical products concluded that most countries had the legal mandate to implement measures to curb substandard and falsified medical products. Still, they were not implemented (13).” (lines 523-530).

***Reviewers comment:***

18. ***Line 462: The US acronym is already used. Please redefine at the beginning of the manuscript. Same suggestion can be addressed for European Union.***

Response: The acronym was redefined for both the United States and European Union in lines 86-88 as shown as “Regulatory processes in the African region are not as well documented as those in the United States Food and Drug Administration (USFDA) and European Union (EU) European Medicines Agency (5).”

***Reviewers comment:***

19. ***Line 468: “LI” please avoid the use of acronyms also in the titles.***
20. ***Line 478: “LT” please avoid the use of acronyms also in the titles.***

Response: Please refer to the response to request 11 above.

***Reviewers’ comment:***

21. ***Line 480: Please rephrase clarifying what it is included what it is not.***

Response: The statement was rephrased to show that only gloves and condoms were tested to verify their safety and performance before marketing authorization. Other medical devices and IVD medical devices were not subjected to testing as part of the conformity assessment to be granted market authorization. The statement was rephrased: " The laboratory testing regulatory function scored high, with 88% for condom and glove testing, as the laboratory was ISO 17025 accredited. However, this score may be due to external body assessments, leading to an established and effective quality management system. Medical devices other than condoms and gloves were not independently verified by laboratory testing based on the risk class of the medical device.” (lines 544-548).

***Reviewers’ comments***

22. ***Line 492: Please also insert the year in the ISO standards.***

***Response:*** The year the ISO standards were issued were included for ISO 15189:2012 and ISO 17025:2017. The statement reads*, “The evaluation can be performed through on-site audits using ISO 15189:2012 and ISO 17025:2017 requirements. Having an ISO accreditation is a good starting point, but more is needed, as anecdotal evidence shows that laboratories with ISO 15189:2012 accreditation failed the WHO audit for IVDs.”* (lines 550-553).

***Reviewers comment***

23. ***Line 495: Please also insert the year in the ISO standard.***

Response: The year of the ISO 15189 standard was added and read as follows, “The evaluation can be performed through on-site audits using ISO 15189:2012 and ISO 17025:2017 requirements. Having an ISO accreditation is a good starting point, but more is needed, as anecdotal evidence shows that laboratories with ISO 15189:2012 accreditation failed the WHO audit for IVDs.” (lines 550-553).

***Reviewers’ comment***

24. ***Line 503: “RI” please avoid the use of acronyms also in the titles.***

Response: Please refer to the response to request 11 above.

***Reviewers’ comment***

25. ***Line 514: “CT” please avoid the use of acronyms also in the titles.***

Response: Please refer to the response to request 11 above.

***Reviewers’ comment:***

26. ***When regulations are cited, it is better to refer directly to the regulation instead of a document reporting the regulation.***

Response: It is unclear where a document reporting the regulation was cited instead of the source. Regulations that were cited were the European Union Medical Devices Regulation (Regulation (EU) 2017/745 of The European Parliament and of the Council on Medical Devices) and In Vitro Diagnostic Medical Devices Regulations (IVDR- Regulation (EU) 2017/746). These regulations are published in the European Journal, the primary source used for citing (European Commission.

3. Regulation (EU) 2017/745 of The European Parliament and of 620 the Council on Medical Devices. Off J Eur Union. 2017;5(8):175. 621

4. European Commission. Regulation (EU) 2017/746 of the European Parliament and of 622 the Council on in vitro diagnostic medical devices. Off J Eur Union. 2017;5(5):117–76.)

Could you kindly indicate where a regulation was cited from a secondary source so I can address it accordingly?

**R*eviewers’ comment:***

27. ***Citations 14 and 15 are the same paper.***

Response: The citation was corrected. The duplicated citation was deleted and replaced with the correct one.

***Reviewers; comment***

28. ***Please check that the citation style is uniform.***

Response: Citations were checked accordingly and corrected using the Vancouver style.

**Reviewer 2:**

Reviewer #2: This is an important paper about the status of medical device regulation in Zimbabwe. The paper requires several revisions.

**R*eviewer Comment:***

1. ***The introduction should be made more concise.***

Response: The introduction was reviewed to make it more concise. Please refer to the introduction in the manuscript (lines 69-221).

***Reviewer comment:***

***2. The methods are unclear. It says that a qualitative study was performed using WHO GBT+ methodology. What does this mean? There is no reference in the methods to a paper or website describing this methodology. Furthermore, the paper should clearly describe the methods without the reader needing to read another paper to understand what was done. It says that regulatory oversight was evaluated from June - August 2022, but what does this mean? Did someone track all medical devices during that time? Did the authors interview people at the NRA? Did they review the documents? Did they review policies? How were the different regulatory functions assessed? How many medical devices were included in this study?***

***It is difficult to review this paper when the reader cannot completely understand the methods and what was done in the study.***

Response: The methodology was reviewed to explain the systematic approach that was used in the selection of the data collection tools, how the data collection site was selected, how participants were selected, how data was collected, coded and analyzed to assign percentage scores for each regulatory function and the overall maturity level of the MCAZ. Please refer to the methodology section in lines 223-321.

***Reviewers’ Comment:***

2. ***Figure 1 does not make sense to me. How did you determine what was estimated versus what was implemented? And if this is real data, it should not appear so perfectly symmetrical / round. Shouldn't there be some variation in different parameters performing better or worse?***

Response: Legend was added to complement the figure in line with the description provided in the methodology. Please refer to lines 358 -361 with the legend in Fig 1.

***Reviewers’ comment:***

3. ***Figure 2 I also do not understand what was measured here and how the authors came up with this data.***

Response: The methodology section was revised to clarify how each regulatory function's implementation quality was calculated using sub-indicators and indicators for each regulatory function. The sum of implemented sub-indicators was used as the numerator and the total sub-indicators as the denominator for each regulatory function. Additionally, an explanation was provided on how the maturity level of the national regulatory authority is determined using the scores for the regulatory functions. A legend was added to Fig. 1 in the manuscript to aid the readers in understanding the figure.
