# Supplementary material for: Assessing operational readiness: Regulatory landscape and compliance in zimbabwe for medical devices and in vitro diagnostic medical devices
Source: PLoS One. 2024 May 16;19(5):e0287495. doi: 10.1371/journal.pone.0287495 (PMC11098427; doi:10.1371/journal.pone.0287495)
Supplement: Attachment — Submitted filename: Response to Reviewers Comments 3 (1).docx [file pone.0287495.s005.docx]

**Major revisions:**

***Reviewers’ Comment:***

1. ***Line 275: “Two participants were interviewed and assisted with information to complete the questionnaires administered by the researcher.” Were only two participants interviewed? Which are the inclusion criteria for which they were chosen?***

***Response:***

Thank you for the comment. Indeed, the inclusion criteria for selecting participants was not defined in the previous version of the manuscript. The sampling method and inclusion criteria were amended as follows, *“The inclusion criteria comprised three main categories: Firstly, officials directly engaged in regulatory decision-making were considered integral to providing insights into the decision-making processes. Secondly, individuals with substantial expertise and experience in health product regulation were targeted, ensuring a wealth of knowledge and a nuanced understanding of the regulatory landscape. Lastly, individuals responsible for critical regulatory functions, such as product registration, licensing, quality control, inspections, pharmacovigilance, and post-marketing surveillance, were included to capture diverse perspectives and experiences within the regulatory domain. Through this careful selection process, the study aimed to gather a well-rounded and insightful perspective on health product regulation from those actively shaping and implementing regulatory policies.” (lines 155-165).*

***Reviewer’s comment***

2. ***Introduction section is still long. The reviewer’s suggestion is to maintain only the information necessary to have an overview of the problem addressed by the manuscript.***

Response: The introduction was reduced by removing some of the content not specific to the Zimbabwean context. (See lines 71-126).

***Reviewer’s comment***

3. ***Grammar revision has been not effective. Here some examples:***

***“Medical devices are essential for the public to meet their highest health standards”instead of “Medical devices are essential for the public to reach the highest health standards.”***

***“Medical Device Regulations are a set of laws and regulations governing the clinical trials,***

***manufacturing, and distribution of medical devices to ensure that they are safe and perform asintended by their manufacturers”Instead of “Medical Device Regulations are a set of laws and regulations governing clinical trials, manufacturing, and distribution of medical devices ensure they are safe and perform as intended by their manufacturers”.***

***“Participation in the study was voluntary” instead of “Participation was voluntary”***

***“The participants were able to stop the interview at any time without any explanation ”instead of “Participants were able to stop the interview at any time without explanation”***

***These examples are not exhaustive.***

Response: Thank you for the comments. The examples were addressed accordingly; see lines 71-72, “*Medical devices and In Vitro Diagnostic (IVD) medical devices are essential for the public to reach their highest health standards.”*

Lines 86-88, “*Medical device and IVD regulations are laws that govern the design and development, manufacturing, clinical trials, and distribution of medical devices. The aim is to ensure that they are safe and perform as intended by their manufacturers (3,4)*.”

Lines 220-221: “*Participation in the study was voluntary. The participants were able to stop the interview at any time without any explanation, if necessary.”*

Furthermore, the manuscript was reviewed extensively to correct the grammatical and typographical errors.

***Minor revisions:***

***Reviewer’s comment***

4. ***Image 1 is low quality. In addition, in the opinion of the reviewer the image does not add any value to the manuscript.***

Response: The image may be of low quality, but it is still valuable as it provides the readers with an overview of the quality of implementation and the maturity level of each regulatory function that applies to medical devices and IVDs' regulatory system. This is the key output of benchmarking conducted by regulatory authorities. Therefore, removing it, as suggested by the reviewer, defeats the whole purpose of conducting the benchmarking. Regarding the quality of the figure, we used the pace application to create an image as required by Plos One journal. Therefore, the authors have decided to keep the image as part of the manuscript.

***Reviewer’s comment***

5. ***Line 117-118:“In 2016, the World Health Organization (WHO) conducted a 117 review to evaluate the availability of medical device regulations in Africa.” Are you sure only Africa was the target area of the study? Probably you are referring to WHO regulatory status desk survey (July 2016 update) and this study was targeted to map all the world status. Please even if this is not the study you have analyzed, in the text which document you are referring to***.

Response: It was a typographical error. The referenced source (<https://www.who.int/teams/health-product-policy-and-standards/assistive-and-medical-technology/medical-devices/regulations>) referred to the global Desk Review of Medical Device Regulatory Systems. The statement was rephrased to read as follows, “In 2016, the World Health Organization (WHO) conducted a review to evaluate the availability of medical device regulations globally.” (line 93).

***Reviewer’s comment:***

6. ***Line 146:” countries of the Surgeons of Central, Eastern and Southern Africa (COSECA)”, the acronym was already defined.***

Response: We amended to state the abbreviation after first being mentioned in line 106 (the amendment is in line 120 and 124).

***Reviewers’ comment***

7. ***Line 228: “The researcher interviewed the staff responsible for the regulations and conducted a desk reviewed the documents.” Please check the grammar or rephrase.***

Response: The statement was rephrased: "*A total of two participants were interviewed. Additionally, an extensive desk review of pertinent documents was conducted to validate the current status of legal provisions, regulations, and guidelines governing the regulatory landscape. This comprehensive approach ensured a nuanced understanding of the regulatory framework within the MCAZ.”*(lines 165-169).

***Reviewer’s comment***

8. ***Line 548: Please insert the year of the standard.***

Response: “The laboratory testing regulatory function for condoms and gloves was above average. This could be due to the ISO 17025:2017 accreditation and assessments by external organizations. Other medical devices and IVDs were not independently verified by laboratory testing based on the risk class of the medical device. Outsourcing laboratory testing is possible; however, laboratories' selection, monitoring, and evaluation should be clearly defined and documented. The evaluation can be performed through on-site audits using ISO 15189:2012 and ISO 17025:2017 requirements*.”* (lines 420 and 425).

***Reviewer’s comment***

9. ***The reviewer suggests to cut all the parts of the manuscript that really do not refer to the main scope and to insert them as supplementary material.***

Response: The introduction was reduced by removing the context of the regulatory system in the United States of America and the European Union, Convergence, Reliance and Recognition Mechanisms and those for the Southern African Development Community region without granular data for Zimbabwe.

***Reviewer’s comment***

10. ***Answer to comment26 of the first review. Reference n.2(GHTF. Global Harmonization Task Force Study Group 1: Definition of the Terms ‘Medical Device’ and ‘In Vitro Diagnostic (IVD) Medical Device.’ Force, Study Gr 1Glob Harmon Task [Internet]. 2012;(Ivd):6. Available from:http://www.imdrf.org/docs/ghtf/final/sg1/technical-docs/ghtf-sg1-n071-2012definition-of-terms-120516.pdf#search=%22ghtf definition ?Medical Device?***

***is not necessary as you can refer directly to MDR 745/2017 and IVDR746/2017 for the definitions of medical device and in-vitro diagnostic medical device.***

***Line numbers refer to the version without track changes.***

Response: Thank you for pointing out where regulations should have been cited directly. However, the Global Harmonization Task Force is the primary source that defines Medical Devices and In Vitro Diagnostic Medical Devices from which regulatory authorities such as the European Union’s MDR 745/2017 and IVDR 746/2017 adopted their definition. The MDR 745/2017 and IVDR746/2017 regulations were published in 2017, which were later compared to the GHTF guidance document published in 2012. In this regard, the authors will maintain this citation and reference as in the previous version of the manuscript.

***Reviewer’s comment***

***Reviewer #2: The authors have made several changes that have improved the paper. However, more changes are needed.***

1. ***The authors refer to Resource Limited Settings (RLS). Please just use the term "LMIC" - low- and middle-income countries, as the other reviewer suggested, as this is the appropriate term. Most countries in Africa are not without resources. There are actually a lot of resources (diamonds, coffee, oil, etc...). The use and corruption of these resources is complicated. So please just stick with LMICs.***

Response: Thank you for the comment and for explaining the rationale of using Low and Middle Income Countries instead of Resource-Limited Settings. The terminology has be amended as requested ( see line 73-74).

***Reviewer’s comment:***

2. ***The other reviewer has complained about too many abbreviations, and there are still too many abbreviations / acronyms. I would limit the paper to 3-5 acronyms and then spell out everything else. The reader cannot keep track of all of the acronyms.***

Response: The abbreviations have been reduced to 5. The following are the abbreviations in the text, IVD, WHO, COSECA, MCAZ, and ISO.

***Reviewer’s comment***

3. ***The introduction is still significantly too long. The authors need to express the same content, but use half as many words. There is a lot of redundant information in the introduction.***

Response: The introduction was reduced by removing the context of the regulatory system in the United States of America and European Union, Convergence, Reliance and Recognition Mechanisms, and from the Southern African Development Community region without granular data for Zimbabwe, but aggregated data without identifying the individual countries was excluded.

**Reviewer’s comment**

4. ***In general, the paper is about a very important topic and should be published. However, the authors need significant help from an experienced author to restructure the paper to make it more readable. In its current form, it is too repetitive in some areas, and then does not give adequate details in the methods and results sections. I would encourage the authors to seek out a global health partner, who has more experience publishing scientific manuscripts to help them with the structure of the paper.***

Response: The manuscript was reviewed by two reputable researchers, as suggested. Their comments and recommendations were considered for the amended submission that was submitted. The definitions for regulatory functions were listed in a table instead of bullet form. Additionally, a list of indicators presented in bullet form was converted into a table, including the description of each indicator. Redundant information in the introduction was removed. The discussion was reduced to avoid repeating the findings reported in the results section. In this section, we compared our findings with other studies that have been conducted. We also indicated new findings and the strengths of our findings.
