# Supplementary material for: Assessing operational readiness: Regulatory landscape and compliance in zimbabwe for medical devices and in vitro diagnostic medical devices
Source: PLoS One. 2024 May 16;19(5):e0287495. doi: 10.1371/journal.pone.0287495 (PMC11098427; doi:10.1371/journal.pone.0287495)
Supplement: Attachment — Submitted filename: Responses to Reviewers.docx [file pone.0287495.s006.docx]

**Reviewer’s comment: Please review your reference list to ensure that it is complete and correct. If you have cited papers that have been retracted, please include the rationale for doing so in the manuscript text, or remove these references and replace them with relevant current references. Any changes to the reference list should be mentioned in the rebuttal letter that accompanies your revised manuscript. If you need to cite a retracted article, indicate the article’s retracted status in the References list and also include a citation and full reference for the retraction notice.**

*Response: The reference list was reviewed. We verified if any of the cited sources were retracted by accessing external websites or databases for the retraction status of references. We searched the references from PubMed, Google Scholar, and the respective publishers' websites, namely the World Health Organization, Global Harmonization Task Force (GHTF), International Medical Devices Regulators Forum (IMDRF), and Regulatory Professionals Affairs Society (RAPS). We looked for any retraction notices or updates. We noted that the web links for references 1, 10, 13,14, 15 and 16 had been changed from the source. We amended the web links with the new links. Additionally, the WHO website was intermittently accessible, which may have made the references from the WHO website inaccessible.*

*Additionally, reference number 23 was added as a supplement to reference 22. Reference 23 is an addendum to reference 22.*

**Review Comments to the Author

Please use the space provided to explain your answers to the questions above. You may also include additional comments for the author, including concerns about dual publication, research ethics, or publication ethics. (Please upload your review as an attachment if it exceeds 20,000 characters)**

**Reviewer #2: This manuscript is greatly improved from the last revision, and it is almost there!

I think the only remaining is that the discussion section is too long, and they are basically re-reporting the results. They need to significantly reduce the discussion section. Instead of restating the results, the authors should just describe how this study fits in with work that has previously need done, and maybe give some details regarding why the regulatory system is so underdeveloped and if there are any plans or what is needed to change this in the future.**

*Response: The discussion was reviewed to remove the results. There was not much literature specific to the Zimbabwean setting; there was only one study by Hubner et al.(* [*https://pubmed.ncbi.nlm.nih.gov/33764886/*](https://pubmed.ncbi.nlm.nih.gov/33764886/) *) that had granular data specific to Zimbabwe to compare with. Other studies by Mwedzi-Dube et al.(* [*https://pubmed.ncbi.nlm.nih.gov/33029353/*](https://pubmed.ncbi.nlm.nih.gov/33029353/) *) and Kniazkov et al. (*[*https://pubmed.ncbi.nlm.nih.gov/33088577/*](https://pubmed.ncbi.nlm.nih.gov/33088577/)*) were for Southern Africa, including Zimbabwe. However, the data was not granular enough to identify where Zimbabwe stood, and the studies were not specific for regulating medical devices and IVDs but health products in general. Therefore, the introduction of the manuscript starts with the statement, “ This is the first study of Zimbabwe’s medical devices and IVD regulations, focussing on all regulatory functions.” (Lines 286-287 of Revised Manuscript with Tracked Changes).*

*The reason why the regulatory system is underdeveloped is stated in lines 287 -294 as follows, “The regulatory system concerning medical devices and IVDs in Zimbabwe is notably deficient due to an underdeveloped legislative framework for regulatory functions. The Medicines and Allied Substances Control Act was enacted in 1969 and only focused on the medicine framework. Subsequent amendments were implemented to include the formulation and implementation of regulation of condoms and gloves. The amendments did not cover all the regulatory functions for an effective regulatory system proportionate to the different health products. This is why the regulatory system for medical devices and IVDs is underdeveloped.”*

*The authors suggested what needs to change based on the findings. Lines 506-540 of the Revised Manuscript with Tracked changes propose changes in the Medicines and Allied Substances Control Act to incorporate the crucial components of the legislative framework as follows,*

*“To effectively regulate medical devices and IVDs in Zimbabwe, The Medicines and Allied Substances Control Act needs to be amended to incorporate key components into the legislative framework:*

*1.	Definition and Classification: Clearly define medical devices and IVDs, establishing a risk-based classification system for regulatory oversight.*

*2.	Regulatory Authority Establishment: Empower the MCAZ with adequate resources and expertise to enforce regulatory requirements effectively.*

*3.	Licensing and Registration: Outline requirements for the licensing or registration of manufacturers, importers, and distributors, ensuring compliance with quality management systems and post-market surveillance obligations.*

*4.	Quality Management Systems: Mandate implementation of quality management systems by manufacturers, adhering to specified standards and guidelines, with regular audits and inspections for compliance verification.*

*5.	Conformity Assessment: Establish procedures for conformity assessment to ensure devices meet regulatory requirements, including pre-market approval, product testing, and clinical evaluations.*

*6.	Post-Market Surveillance: Mandate post-market surveillance systems for monitoring device safety and performance, with provisions for adverse event reporting, recalls, and periodic re-evaluations.*

*7.	Labeling and Instructions for Use: Require clear labeling and instructions for use to ensure safe and effective device utilization by healthcare professionals and end-users.*

*8.	Capacity Building and Training: Include provisions for capacity building and training initiatives to enhance regulatory capacity among stakeholders, including regulatory authorities, healthcare professionals, and industry representatives. The competence framework must be put in place that includes proficiency in scientific and health concepts, encompassing the understanding and applying evolving basic and translational science, regulatory science, and public health principles to enhance healthcare product development, evaluation, and oversight (21–23).*

*9.	International Harmonization: Align legislative provisions with international standards and guidelines to facilitate trade and harmonization efforts, potentially adopting regulations developed by the International Medical Devices Regulators Forum and WHO.*

*Incorporating these components into the legislative framework enables Zimbabwe to establish a robust regulatory system for medical devices and IVDs, ensuring their safety, quality, and effectiveness in healthcare delivery.”*
